# Retraction

**DOI:** 10.1002/joa3.12864

**Published:** 2023-05-03

## Abstract

Retraction: Hasegawa K, Fukuoka Y, Ohno S, Horie M, Tada H. Computerized misinterpretation of QT interval in 12‐lead electrocardiogram and its clinical consequences: a case of recurrent syncope. *J Arrhythmia*. 2023;39:227–30. https://doi.org/10.1002/joa3.12817

The above article from *Journal of Arrhythmia*, published online on January 30, 2023, in Wiley Online Library (wileyonlinelibrary.com) and in Volume 39, pp. 227–230, has been retracted by agreement between the authors, the journal Editor in Chief Kazuo Matsumoto and John Wiley & Sons Ltd. The retraction has been agreed due to duplication with a previously published article from the same group of authors.

Retraction: Hasegawa K, Fukuoka Y, Ohno S, Horie M, Tada H. Computerized misinterpretation of QT interval in 12‐lead electrocardiogram and its clinical consequences: a case of recurrent syncope. *J Arrhythmia*. 2023;39:227–30. https://doi.org/10.1002/joa3.12817

The above article from *Journal of Arrhythmia*, published online on January 30, 2023, in Wiley Online Library (wileyonlinelibrary.com) and in Volume 39, pp. 227–230, has been retracted by agreement between the authors, the journal Editor in Chief Kazuo Matsumoto and John Wiley & Sons Ltd. The retraction has been agreed due to duplication with a previously published article from the same group of authors.

